# “Stay-at-Home” Lifestyle Effect on Weight Gain during the COVID-19 Outbreak Confinement in China

**DOI:** 10.3390/ijerph18041813

**Published:** 2021-02-12

**Authors:** Qi Zhu, Min Li, Yu Ji, Youpeng Shi, Jie Zhou, Qianyue Li, Ruoyu Qin, Xun Zhuang

**Affiliations:** 1 School of Public Health, Nantong University, Nantong 226019, China; zgjsntzq@126.com (Q.Z.); 1917310012@stmail.ntu.edu.cn (M.L.); 2 Xinglin College, Nantong University, Nantong 226008, China; JYhxq991011@163.com (Y.J.); 18068110729@163.com (Y.S.); zj462336041@163.com (J.Z.); zdpzg96@163.com (Q.L.); 15152885351@139.com (R.Q.)

**Keywords:** COVID-19, “stay-at-home” lifestyle, food intake, physical activity, weight gain

## Abstract

In February 2020, a novel coronavirus (SARS-COV2) broke out in Wuhan city of China. The Chinese government decisively imposed nationwide confinement. This study comprised a structured, online questionnaire, based on 40 items inquiring about socio-demographic information and anthropometric data (reporting weight and height), as well as changes in food intake, physical activity, and sleep during the COVID-19 outbreak. Questionnaires were distributed to residents of Jiangsu and other provinces from 29 March to 5 April. A total of 889 respondents were included, aged between 16 and 70 years (61% females). There was a significant increase in total food intake by 9.8% and a slight increase by 29.2% of respondents, and a significant decrease in physical activity by 31.5% and a slight decrease by 23.4% of respondents, especially in snacks and drinks, and outdoor activities. The rate of weight gain in the total population was 30.6% and the average weight gain was 0.5 ± 2.8 kg. The main factors contributing to weight gain were increased food intake and reduced physical activity. Additionally, normal-weight people were more likely to gain weight than people with overweight/obesity during the COVID-19 confinement. This study provided a good warning and educational reference value on lifestyle changes during the COVID-19 confinement.

## 1. Introduction

A novel coronavirus disease, later abbreviated to COVID-19 by World Health Organization (WHO) [1], is a viral infectious respiratory disease, which can spread quickly through droplets formed by water and various inclusions, and the droplets can be generated while talking, breathing, coughing, or sneezing [2]. It was first seen in Wuhan city, Hubei province, China, in December 2019 [3]. Soon after, WHO declared the COVID-19 outbreak a public health emergency of international concern on 30 January [4] and assessed COVID-19 as a pandemic on 11 March [5].

As definitive vaccines and cures for COVID-19 are unlikely to be identified soon [6], social isolation seems to be the most effective strategy to control the COVID-19 outbreak [7]. This strategy will be effective for flattening the curve of new infections due to human-to-human transmission, limiting morbidity, mortality, and the ensuing surge of demand on the healthcare system. So, the public has been advised to reduce movement and stay at home as a basic means of limiting people’s exposure to the virus. Health authorities, including the National Health Commission of the People’s Republic of China [8], WHO [9], and U.S. Centers for Disease Control and Prevention (CDC) [10], have issued safety recommendations for taking related precautions to reduce exposure to and transmission of the virus. In fact, throughout the COVID-19 epidemic, the Chinese people have shown unprecedented solidarity, actively cooperating with the government’s measures and calls, greatly reducing the need to go out of the house, and almost entering the mode of the whole people staying at home. This “lockdown” strategy has proven effective for containing the COVID-19 outbreak in China, also limiting the exportation of infected cases outside the country [11,12]. A similar situation followed in the United States. Over 90% of U.S. adult residents were confined to their homes, with restaurants, shops, schools, and workplaces shut down to prevent disease spread [13].

Nevertheless, the prolonged home life may unintentionally lead to increased sedentary behaviors, such as spending excessive amounts of time sitting, reclining, or lying down for screen activities (playing games, watching television, using mobile devices), reducing regular physical activity (thus lowering energy expenditure), or indulging in excessive food intake that, consequently, causes an increased risk for and potential worsening of chronic health conditions [14]. Some of the most important undesirable consequences of prolonged staying at home, such as physical inactivity, weight gain, behavioral addiction disorders, insufficient sunlight exposure, and social isolation, were critically addressed by some scholars [15]. In particular, weight gain during adulthood was associated with a significantly increased risk of major chronic diseases and decreased odds of healthy aging [16].

During the global pandemic COVID-19 lockdown, studies reported weight gain in different countries. For instance, the perception of weight gain was observed in 48.6% of the population in an Italian survey [17]. A study of 173 people found that 22% of residents gained 2.3–4.5 kg during self-quarantine in the United States, and risk factors for weight gain were inadequate sleep, snacking after dinner, lack of dietary restraint, eating in response to stress, and reduced physical activity [18]. A study from Poland (*n* = 1.097) found that almost 30% of subjects experienced weight gain (3.0 ± 1.6 kg), and overweight, obese, and older subjects tended to gain weight more frequently during COVID-19 lockdown [19]. A cohort analysis during 49 days of lockdown in India showed a trend toward weight gain seen in 40%, with 16% of the population experiencing a 2.1–5.0 kg weight increase, and it may increase the risk of type 2 diabetes mellitus [20].

To comprehensively understand and analyze the impact of the domestic lifestyle due to the COVID-19 outbreak on the diet, exercise, and sleep of the Chinese people, we conducted this survey. It is worth mentioning that during the period of this investigation the epidemic in China was controlled. However, at the same time, the rest of the world was in the midst of a rapid outbreak, people were living in dire conditions, and the global hazards caused by the COVID-19 pandemic were far from over. Therefore, this survey will have a good reference value for countries that adopt ‘limiting to go out’ measures and also provide a basis for the active intervention of residents’ quality of life in similar public health emergencies in the future.

## 2. Materials and Methods

### 2.1. Study Design and Population

In order to cope with the outbreak of the COVID-19 epidemic, this survey needed to evaluate the lifestyle of the population in a short period of time. So, a rapid epidemiological assessment was adopted. Due to the “stay at home” regulation of the Chinese people, the on-site survey could not be carried out and an online questionnaire could only be chosen. Accordingly, a cross-sectional sample of 1040 Chinese residents aged 16–70 years was recruited online via our research group from 29 March to 5 April. The gender, age, urban and rural areas, family income, educational level, and so on of the collected population were taken into account. These people were all voluntary participants, which greatly reduced the non-response bias that often exists in the on-site survey. However, as our research group is located in the Jiangsu Province, according to the survey results, residents in the Jiangsu region accounted for the largest proportion (68.46%), followed by nearby places such as Shanghai City (8.17%) and Zhejiang Province (4.23%), and the remaining 26 provinces together accounted for 19.14%. Therefore, this survey may be not a good representation of all Chinese residents. However, under such urgent conditions at that time, it more or less reflected the lifestyle of the residents in the most economically developed and ideologically active regions of China for the first time.

All the participants were informed of the purpose and procedures of the study and participated voluntarily and anonymously. This study was approved by the ethical review committee of Nantong University (Ethics number: 202062).

### 2.2. Survey Development and Content

A very popular professional questionnaire platform, named ‘Wen Juan Xing’ in Chinese or ‘Sojump’ in English, was used to develop the questionnaire survey system. Through several simulation tests, the content of the questionnaire was continuously improved. Key content areas were developed by applying the socio-ecological model, including responders’ demographics, current diet, physical activity, and sleep behaviors, as well as changes before and during the outbreak. It should be noted that this study was a cross-sectional survey that collected the data at the time of the outbreak, while the data before the COVID-19 outbreak were recorded by the responders through their recollections. For those subjective qualitative questions, it was reported using a five-point Likert type scale, ranging from “a lot less” (score = 5) to “no change” (score = 3) to “a lot more” (score = 1). This self-designed questionnaire consisted of four parts, as follows.

#### 2.2.1. Sociodemographic Information

Gender, date of birth, living in the urban or rural areas during the epidemic, family per capita monthly income, resident population, educational background, body height, and body weight were included.

#### 2.2.2. Diet

(1) Change of dietary intake before and during the epidemic, including 10 categories: total intake, staple foods, vegetables, dairies, legumes or products, livestock/poultry meats, fished or fishery products, eggs, fruits, snacks/beverages;
(2) Six psychological emotions on dietary behavior before and during the epidemic: dysphoria, anxiety, sadness, nervous, loneliness, depression; and
(3) Dietary restriction, overeating, and storing food behavior during the epidemic.

#### 2.2.3. Physical Activity

(1) Physical activity time, sedentary time, outdoor exercise time, and average daily steps before and during the epidemic;
(2) Time spent using mobile phones and computers and doing housework during the epidemic; and
(3) Weekly frequency of vigorous exercise and moderate exercise, as well as going out and walking during the epidemic.

#### 2.2.4. Sleep

(1) The time to go to bed, fall asleep, and get up; the length of sleep; the quality of sleep before and during the epidemic;
(2) Factors affecting sleep during the epidemic: disturbance in respiration, cough or snore, feeling cold or hot, having a nightmare; pain or ache; and
(3) Feeling sleepy and undynamic during the epidemic.

Moreover, for every certain number of questions in the questionnaire, a quality control question was inserted to ask the respondents to choose the specified answer, to find the respondents who give a random answer. Test-retest (one week) reliability was assessed via bivariate Pearson correlation (*n* = 100) and correlation coefficient range = 0.76–0.98 (strong reliability).

### 2.3. Statistical Analysis

The data from the questionnaire survey were exported to the SPSS 22.0 statistical software for data logical verification and statistical analysis. 

Firstly, the samples that were randomly answered in the quality control questions were eliminated. The samples with missing or implausible data such as a self-reported body weight of 10 kg or 200 kg were excluded. Illogical or paradoxical samples were also removed, e.g., those whose birth date was later than the time of the survey; those with contradictory answers to questions, such as respondents who reported significant weight gain, but actually lost weight after calculation; and so on. Therefore, these exclusion criteria had few effects on the external validity while improving the accuracy of the survey results.

In this study, the quantitative data were expressed as mean value ± standard deviation (mean ± SD). A paired *t*-test was used to compare the measurement data (body weight, step number per day, outdoor activities’ duration, sedentary duration, actual sleep duration, the time it takes to fall asleep) before and after the COVID-19 outbreak. Analysis of variance (ANOVA) was used for comparison of weight gain between groups (gender, age, type of residence, household income, permanent resident population, educational background, and overweight or obesity, as well as changes in total food intake, physical activity, and sleep). The confounders (gender, age, BMI, step number, outdoor activities’ duration, sedentary duration, and actual sleep duration) were adjusted for by analysis of covariance (ANCOVA). The count data were expressed by frequency (n) and percentage (%), and Pearson’s *χ*^2^ test was used for comparison between groups. The linear regression equation between the total score of the three main factors (changes in total food intake, physical activity, and whether overweight/obesity before the outbreak) and weight gain was calculated. Multiple linear regression modeling was used to analyze the interactions between the changes in total food intake, changes in physical activity, and the pre-outbreak overweight/obesity effect on weight gain. A *p*-value < 0.05 was considered statistically significant.

## 3. Results

Excluding the invalid and illogical data, the final number of respondents was 889. Among them, 347 (39%) were males and 542 (61%) were females. The ages ranged from 16–70 years old and the average was 31.8 ± 11.4 years. For residence type, 661 (74.4%) people lived in urban areas and 228 (25.6%) people lived in rural areas. During the COVID-19 outbreak, 607 (68.3%) people lived in Jiangsu province, 71 (8.0%) people lived in Shanghai city, 36 (4.0%) people lived in Zhejiang province, and 175 (19.7%) people lived in the other 31 provinces except Hainan, Ningxia, Hong Kong, Macao, and Taiwan. The education background was 78 (8.8%) completed junior high school, 116 (13.0%) completed senior high school, 618 (69.5%) completed bachelor’s degree, 57 (6.4%) completed master’s degree, and 20 (2.2%) completed doctoral degree. Per capita monthly household income was 211 (23.7%) people with less than 3000 yuan ($462), 252 (28.3%) people with 3000–6000 yuan ($462~924), 211 (23.7%) people with 6000–10,000 yuan ($924~1540), 143 (16.1%) people with 10,000–20,000 yuan ($1540~3080), and 72 (8.1%) people with more than 20,000 yuan ($3080). Permanent resident population was 1 population, 15 (1.7%); 2 population, 73 (8.2%); 3 population, 355 (39.9%); 4 population, 228 (25.6%); 5 population, 155 (17.4%); 6 population, 40 (4.5%); and 6 population and above, 23 (2.6%).

### 3.1. Changes in Food Intake, Physical Activity, and Sleep Compared to before the COVID-19 Outbreak 

Changes in food intake, physical activity, sleep duration, and sleep quality were rated on a scale of five compared to before the outbreak. The detailed results are shown in Table 1. The results of overeating for psychological reasons are shown in Table 2. The results of physical activity during the outbreak are shown in Table 3. The changes in body weight, steps, outdoor activities’ duration, sedentary duration, and sleep duration during the outbreak compared to before are shown in Table 4. In general, due to the “stay-at-home” lifestyle, there was a certain increase in food intake, a significant decrease in physical activity, especially outdoor exercise, and a slight increase in body weight, sedentary duration, and sleep duration during the COVID-19 outbreak.

### 3.2. Analysis of Factors Affecting Weight Gain during the “Stay-at-Home” Lifestyle Caused by COVID-19 Outbreak

The proportion of respondents who gained weight (>1 kg) during the COVID-19 outbreak was 30.6% (34% male, 28.0% female, no statistical difference, *p* = 0.074), with an average weight gain of 0.5 ± 2.8 kg. Firstly, through the one-way analysis of variance (ANOVA), it was found that changes in total food intake (*p* = 0.000) and physical activity (*p* = 0.020), as well as whether overweight/obesity before the COVID-19 outbreak (*p* = 0.004), had a significant effect on weight gain due to the “stay at home” lifestyle. After further adjusting for potential confounding factors by using the analysis of covariance (ANCOVA), the above three variables still had significant effects on weight gain, as shown in Table 5.

Other demographic indicators including gender (*p* = 0.254), age (*p* = 0.302), urban or rural residents (*p* = 0.312), per capita monthly household income (*p* = 0.408), permanent resident population (*p* = 0.384), and educational background (*p* = 0.390), as well as changes in sleep duration (*p* = 0.416) and sleep quality (*p* = 0.062) had no significant effect on weight gain. 

Secondly, according to the effect of the three main factors (changes in total food intake and physical activity, whether overweight/obesity before the outbreak) on weight gain, each individual was assigned a score, and then the sum was added to calculate the total score, so that the linear regression equation between the total score of the three main factors and weight gain was calculated (Table 6).

**Figure 1:**
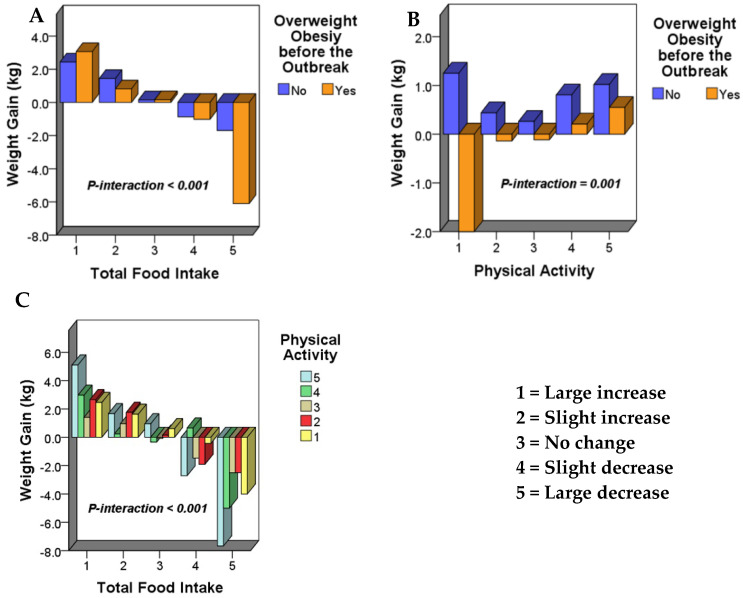
(**A**) The interaction between changes in total food intake and whether overweight/obesity before the outbreak on weight gain. (**B**) The interaction between changes in physical activity and whether overweight/obesity before the outbreak on weight gain. (**C**) The interaction between the changes in total food intake and physical activity on weight gain.

Furthermore, the interaction analysis of the three main factors that significantly affect weight gain during the “stay-at-home” lifestyle was carried out, and it was found that:

(1) “Changes in total food intake * whether or not overweight/obesity before the outbreak” had a significant interaction effect on weight gain (*p*-interaction < 0.001) (Figure 1A).
(2) “Changes in physical activity * whether or not overweight/obesity before the outbreak” had a significant interaction effect on weight gain (*p*-interaction = 0.001) (Figure 1B).
(3) “Changes in total food intake * changes of physical activity” had a significant interaction effect on weight gain (*p*-interaction < 0.001) (Figure 1C).

Lastly, all variables correlated with weight gain during the COVID-19 outbreak are listed in Table 7, from high to low in terms of correlation coefficients. The unlisted variables, including demographic indicators and changes in sleep duration, were not correlated with weight gain.

## 4. Discussion

The purpose of this study was to assess the immediate changes in food intake, physical activity, and sleep quality in Chinese residents during the initial period of the COVID-19 outbreak. We found that Chinese residents had more food intake, were less active, were more sedentary, and gained weight during the initial period of the COVID-19 outbreak compared with before the restrictions. These changes did not differ in demographic indicators such as gender, age, urban or rural areas, household income, educational background, and so on. The largest changes reported were the increased intake of snacks/beverages and decreased outdoor activity. These findings confirm speculations that pandemic-related confinements were unfavorably related to lifestyle. This observation had triangulated support from quantitative (descriptive and correlational) and qualitative (contextual) evidence.

The COVID-19 outbreak is a classic public health emergency. A review reported negative psychological effects after isolation, including post-traumatic stress symptoms, confusion, and anger [21]. According to a survey of 1210 respondents in 194 Chinese cities during the initial period of the COVID-19 outbreak, 84.7% of the population spent 20–24 h per day at home, and a considerable number of people had psychological problems of varying degrees: 8.1% reported moderate to severe stress, 16.5% reported moderate to severe depression, 28.8% reported moderate to severe anxiety, and 75.2% were concerned about family exposure to COVID-19 [22]. Several pieces of research revealed that these psychological problems can affect the way people live, more or less [23]. Increased stress, anxiety, and boredom can lead to higher energy intake, sleep disorders, and less exercise, leading to the risk of weight gain [13,24]. 

Similarly, in this study, we found that about 40% of the respondents had different degrees of increase in total food intake, and the increase in food intake was probably associated with psychological factors. The rapid outbreak of COVID-19, its high infectivity, and long epidemic period, together with the various lockdown measures adopted, constitute a stressor for the general public, making some especially vulnerable groups more anxious and depressed than before the outbreak. Either acute mild stress or prolonged chronic stress can influence our appetite, including our desire to eat and the types of food we are likely to choose [25]. Studies have shown that not only in the hours to days after the onset of an ongoing stressful event but also in chronic stress and mood disorders, glucocorticoids in the bloodstream are elevated [26,27], which act on the hypothalamus to stimulate appetite [28]. Experimental animal studies also have shown that chronically stressed animals prefer high-calorie foods, and a junk food diet or a stress-induced ice-cream binge may actually alleviate the symptoms of stress [29,30]. This study found that about a quarter of the residents suffer from psychological problems including loneliness, agitation, anxiety, tension, sadness, depression, and staying at home all day with nothing to do, which may lead them to eat more food. Especially, the increase of snack and beverage intake can bring the excess of energy and cause weight gain. This study revealed that 30.6% of respondents gained more than 1 kg during their “stay at home”, 17.4% gained more than 3 kg, and 7.3% gained more than 5 kg. Although the average weight gain of 0.5 ± 2.8 kg in this study was small (which was similar to the previous study on holiday weight gain [31]), the standard deviation of 2.8 kg was large, which means the weight gain of some individuals was relatively large, and the maximum weight gain was 20 kg. As evident from previous research, small changes in body weight and fat accumulation in relatively short periods can become permanent and lead to substantial weight gain over time [32,33,34], and changes in eating behavior are likely the main driver toward energy surplus [35]. Similarly, in this Chinese study, the increase in food intake was the most correlated with weight gain among all the factors, with the increase in staple food, snacks/beverages, and livestock and poultry intake ranking the top three. Another domestic study also found that changes in diet and physical activity are independent risk factors for weight gain during the COVID-19 outbreak [36]. At the same time, current food purchasing trends in the USA also clearly indicated that households are stocking up on shelf-stable, ultra-processed, energy-dense comfort foods such as potato chips, popcorn, chocolate, ice cream, and alcohol [37]. In contrast, during COVID-19 confinement in Spain and Italy, respondents adopted healthier eating habits/behaviors, as reflected in higher adherence to the Mediterranean diet [19,38]. 

In terms of energy consumption, due to the suspension of work and production, as well as the closure of fitness venues and public playgrounds, people’s exercise during the epidemic period was significantly reduced, replaced by more screen time. This survey found that more than half of the respondents had reduced physical activity during the COVID-19 outbreak compared with before (with a “significant decrease” accounting for 31.5%), including decreased outdoor activity, decreased step number, and increased sedentary time, which were also significantly associated with weight gain. Studies have shown that the abrupt interruption of physical exercise and prolonged inactivity may promote many adverse health changes, including the development of insulin resistance, muscle atrophy, bone loss, decreased aerobic capacity, increased blood pressure and heart frequency, fatty liver disease, nonalcoholic steatohepatitis, and dyslipidemia, as well as a higher risk of collapsing upon resuming exercise [39]. Therefore, important biological and metabolic adaptations may lead to significantly increased risks of osteoporosis [40], diabetes [41], cardiovascular disease [42], cancer [43], and dementia [44]. Overall, the increased burden of disease due to physical inactivity can increases the risk of all-cause death in the general population by nearly 24% [45]. Although our study only analyzed the risk of weight gain, overweight and obesity are accompanied by a kaleidoscope of metabolic derangements, ultimately increasing the risk of various chronic diseases [46].

Contrary to previous studies that people with overweight and obesity were more likely to gain weight [17,35], this study yielded an unusual finding, which was that people with overweight and obesity gained significantly less weight than normal people during the COVID-19 outbreak and people with normal weight before the outbreak had higher food intake and less physical activity during the outbreak than those with overweight or obesity. We speculated that people with overweight and obesity had been accustomed to the unhealthy lifestyle of eating more and exercising less before, so the change was not obvious during the outbreak, while normal-weight people were more likely to passively change from the healthy lifestyle to unhealthy, leading to weight gain. This intriguing finding also suggests that people of normal weight should pay more attention to preventing and controlling their weight gain during passive home isolation. 

Moreover, the three factors most significantly associated with weight gain, namely, increased food intake, reduced physical activity, and non-overweight and obesity before the outbreak were analyzed through interaction analysis and multiple regression analysis. It was found that when these three main factors were combined, respondents had a higher risk of weight gain. Additionally, while this study found a slight increase in “sleep time” and “sleep quality”, there was no correlation with weight gain. But another domestic study found that after the outbreak of COVID-19, residents’ sleep quality declined, with an obvious sleep disorder [47]. 

Certainly, in addition to the weight gain, there were also some harmful health problems that were not involved in this study, such as behavioral addiction disorders, inadequate sunshine exposure, and social isolation [14]. In particular, we also noticed a few studies that provided insights into the positive impacts of the pandemic, for example, some families increased healthy hobbies or activities like arts, crafts, puzzles, and games [48]. Lastly, several limitations of this study need to be noted. Firstly, as for food intake and physical activity, the self-subjective report used in this study did not make a strict quantitative survey, so the actual results would be biased. Secondly, due to the cross-sectional design, causality cannot be inferred. These will be further improved in future studies. Thirdly, the respondents in this study were mainly in the area of Jiangsu, Zhejiang, and Shanghai, while for Hubei province, which had the worst outbreak of COVID-19, the lockdown was more stringent and lasted longer, potentially causing more health problems. Hence, our data need to be confirmed and investigated in future, more extensive population studies, and we also look forward to relevant studies from other research groups. 

## 5. Conclusions

This study provided data on lifestyle changes during the COVID-19 lockdown about Chinese residents for the first time. There was an increase in total food intake by 39% of respondents, especially in snacks and drinks. The major psychological factors leading to the increase in food intake were loneliness and anxiety. There was a decrease in physical activity by 54.9% of respondents, especially in outdoor activities. The rate of weight gain in the total population was 30.6% and the average weight gain was 0.5 ± 2.8 kg. The main factors contributing to weight gain were increased food intake and reduced physical activity, and normal-weight people were more likely to gain weight than overweight or obese people during the “stay at home” lifestyle caused by the COVID-19 outbreak. Furthermore, the three main factors associated with weight gain interact with each other and can have a synergistic effect on weight gain. 

## Figures and Tables

**Table 1:** Changes in food intake, physical activity, and sleep compared to before the COVID-19 outbreak.

	**Large Increase**	**Slight Increase**	**No Change**	**Slight Decrease**	**Large Decrease**
Total food intake	87 (9.8)	260 (29.2)	428 (48.1)	96 (10.8)	18 (2.0)
Snacks and drinks intake	104 (11.7)	263 (29.6)	378 (42.5)	65 (7.3)	79 (8.9)
Fruits intake	84 (9.4)	303 (34.1)	385 (43.3)	86 (9.7)	31 (3.5)
Vegetable intake	81 (9.1)	228 (25.6)	502 (56.5)	63 (7.1)	15 (1.7)
Eggs intake	75 (8.4)	275 (30.9)	494 (55.6)	34 (3.8)	11 (1.2)
Livestock/poultry meat intake	66 (7.4)	280 (31.5)	418 (47.0)	88 (9.9)	37 (4.2)
Dairy intake	62 (7.0)	191 (21.5)	497 (55.9)	91 (10.2)	48 (5.4)
Staple food intake	60 (6.7)	175 (19.7)	531 (59.7)	97 (10.9)	26 (2.9)
Aquatic products intake	48 (5.4)	266 (29.9)	441 (49.6)	85 (9.6)	49 (5.5)
Legumes intake	36 (4.0)	250 (28.1)	526 (59.2)	55 (6.2)	22 (2.5)
Physical activity	31 (3.5)	111 (12.5)	259 (29.1)	208 (23.4)	280 (31.5)
Sleep duration	169 (19.0)	205 (23.1)	392 (44.1)	75 (8.4)	48 (5.4)
	**Significantly Better**	**Slightly Better**	**No Change**	**Significantly Worse**	**Slightly Worse**
Sleep quality	96 (10.8)	111 (12.5)	533 (60.0)	110 (12.4)	39 (4.4)

The numbers in the table are frequencies and the numbers in brackets are percentages.

**Table 2:** Overeating for psychological factors.

Overeating Because of	Completely Consistent	Basically Consistent	Basically Not Consistent	Completely Not Consistent
Loneliness	34 (3.8)	253 (28.5)	362 (40.7)	240 (27.0)
Agitation	31 (3.5)	225 (25.3)	375 (42.2)	258 (29.0)
Anxiety	25 (2.8)	192 (21.6)	401 (45.1)	271 (30.5)
Tension	20 (2.2)	146 (16.4)	428 (48.1)	295 (33.2)
Sadness	19 (2.1)	134 (15.1)	440 (49.5)	296 (33.3)
Depression	14 (1.6)	190 (21.4)	391 (44.0)	294 (33.1)

The numbers in the table are frequencies and the numbers in brackets are percentages.

**Table 3:** Physical activity during the “stay-at-home” lifestyle.

Day	How Many Days a Week Do Strenuous Physical Activity	How Many Days a Week Do Moderate Physical Activity	How Many Days a Week Go for a Walk (10 min or More at a Time)
0	399 (44.9)	216 (24.3)	241 (27.1)
1	231 (26.0)	206 (23.2)	144 (16.2)
2	129 (14.5)	174 (19.6)	172 (19.3)
3	50 (5.6)	107 (12.0)	113 (12.7)
4	29 (3.3)	59 (6.6)	65 (7.3)
5	24 (2.7)	53 (6.0)	63 (7.1)
6	10 (1.1)	15 (1.7)	23 (2.6)
7	17 (1.9)	59 (6.6)	68 (7.6)

The numbers in the table are frequencies and the numbers in brackets are percentages.

**Table 4:** Changes in body weight, step number, outdoor activities ‘duration, sedentary duration, and sleep duration during the COVID-19 outbreak compared to them before.

Numerical Continues Variables	Before the Outbreak	During the Outbreak	Difference Value of during and before	Paired *t*-Test *p-*Value
Body weight (kg)	61.9 ± 11.6	62.4 ± 11.6	0.5 ± 2.8	<0.001
Step number per day	6427 ± 4374	2714 ± 3542	−3713 ± 4450	<0.001
Outdoor activities duration (min/d)	48.1 ± 51.6	22.8 ± 38.0	−25.3 ± 48.7	<0.001
Sedentary duration (h/d)	5.3 ± 2.7	6.6 ± 3.1	1.3 ± 2.6	<0.001
Actual sleep duration (h/night)	7.6 ± 2.9	8.0 ± 1.4	0.5 ± 1.3	<0.001
The time it takes to fall asleep (min)	23.5 ± 24.7	31.9 ± 38.4	8.5 ± 28.9	<0.001

Variables are expressed as mean value ± SD.

**Table 5:** Changes in total food intake and physical activity compared to before the outbreak, and pre-outbreak overweight/obesity effect on weight gain.

Total Food Intake	Weight Gain ^1^ (kg)	Whether Overweight/Obese before the Outbreak	Weight Gain ^2^ (kg)	Physical Activity	Weight Gain ^3^ (kg)
Large increase	2.55 ± 3.97	Normal weight	0.68 ± 2.37	Large increase	0.10 ± 4.77
(*n* = 87)		(*n* = 661)		(*n* = 31)	
Slight increase	1.31 ± 2.40	Overweight/obesity	0.07 ± 3.61	Slight increase	0.31 ± 2.66
(*n* = 260)		(*n* = 228)		(*n* = 111)	
No change	0.16 ± 1.96	***p*-Value for ANCOVA**		No change	0.16 ± 2.38
(*n* = 428)		^1^*p*-Ancova < 0.001 ^2^ *p*-Ancova < 0.001 ^3^ *p*-Ancova = 0.024		(*n* = 259)	
Slight decrease	−0.92 ± 2.97			Slight decrease	0.66 ± 2.27
(*n* = 96)				(*n* = 208)	
Large decrease	−4.14 ± 3.76			Large decrease	0.90 ± 3.08
(*n* = 18)				(*n* = 280)	

Weight gain is expressed as mean value ± SD; (*n*) sample size. ^1^ Adjusted for gender, age, BMI before the outbreak, difference value of steps, outdoor activities’ duration, sedentary duration, actual sleep duration between during and before the outbreak. ^2^ Adjusted for gender, age, difference value of steps, outdoor activities’ duration, sedentary duration, actual sleep duration between during and before the outbreak. ^3^ Adjusted for gender, age, BMI before the outbreak, difference value of actual sleep duration between during and before the outbreak.

**Table 6:** Score assignment of the three main factors on weight gain and linear regression equation calculation between the total score of the three main factors and weight gain.

Main Factors on Weight Gain	Score Assignment				
	Large Increase	Slight Increase	No Change	Slight Decrease	Large Decrease
Total food intake	1	2	3	4	5
Physical activity	5	4	3	2	1
Overweight/obesity before the outbreak	No	Yes			
	1	2			
**Total Score of the Main Factors**			**Weight Gain (kg)**		
Min = 3, Max = 12, Mean = 6.25, SD = 1.56			Min = −16.0, Max = 20.0, Mean = 0.53, SD = 2.75		
**Linear Regression Equation of Weight Gain and Total Score of the Main Factors**					
* Weight gain (kg) = −0.58 * Total score + 4.148, *p*-coefficient < 0.001					

* The linear regression equation means that for every one-point decrease in the total score of the three main factors, the bodyweight of the respondent increased by 0.58 kg.

**Table 7:** Variables correlated with weight gain, listed in order by the correlation coefficient.

Variables Correlated with Weight Gain	Correlation Coefficient *	*p*-Coefficient
Increase in total food intake *	0.406	<0.001
Staple food intake	0.344	<0.001
Snacks and drinks intake	0.259	<0.001
Livestock/poultry meat intake	0.242	<0.001
Eggs intake	0.236	<0.001
Fruits intake	0.219	<0.001
Dairy intake	0.207	<0.001
Aquatic products intake	0.158	<0.001
Vegetable intake	0.130	<0.001
Legumes intake	0.116	<0.001
Increased food intake for psychological factors *		
Agitation	0.168	<0.001
Loneliness	0.156	<0.001
Tension	0.122	<0.001
Anxiety	0.117	<0.001
Depression	0.108	<0.001
Sadness	0.106	0.001
BMI before the outbreak	−0.161	0.001
Decreased physical activity *	0.102	0.002
Decreased step number *	0.095	0.005
Decreased outdoor activity *	0.091	0.006
Increased sedentary time *	0.075	0.025

* All compared to before the COVID-19 outbreak.

## Data Availability

Access to study data can be provided by the first author (Qi Zhu) upon reasonable request after the study team has completed planned data analyses.
